# Development and Validation of a Taxonomy for Characterizing Measurements in Health Self-Quantification

**DOI:** 10.2196/jmir.6903

**Published:** 2017-11-03

**Authors:** Manal Almalki, Kathleen Gray, Fernando Martin-Sanchez

**Affiliations:** ^1^ Health and Biomedical Informatics Centre Melbourne Medical School The University of Melbourne Melbourne Australia; ^2^ Faculty of Public Health and Tropical Medicine Health Informatics Dept. Jazan University Jazan Saudi Arabia; ^3^ E-Health Research Unit National Institute of Health Carlos III Madrid Spain

**Keywords:** health, self-management, self-experimentation, wearables, quantified self, taxonomy, classification

## Abstract

**Background:**

The use of wearable tools for health self-quantification (SQ) introduces new ways of thinking about one’s body and about how to achieve desired health outcomes. Measurements from individuals, such as heart rate, respiratory volume, skin temperature, sleep, mood, blood pressure, food consumed, and quality of surrounding air can be acquired, quantified, and aggregated in a holistic way that has never been possible before. However, health SQ still lacks a formal common language or taxonomy for describing these kinds of measurements. Establishing such taxonomy is important because it would enable systematic investigations that are needed to advance in the use of wearable tools in health self-care. For a start, a taxonomy would help to improve the accuracy of database searching when doing systematic reviews and meta-analyses in this field. Overall, more systematic research would contribute to build evidence of sufficient quality to determine whether and how health SQ is a worthwhile health care paradigm.

**Objective:**

The aim of this study was to investigate a sample of SQ tools and services to build and test a taxonomy of measurements in health SQ, titled: the classification of data and activity in self-quantification systems (CDA-SQS).

**Methods:**

Eight health SQ tools and services were selected to be examined: Zeo Sleep Manager, Fitbit Ultra, Fitlinxx Actipressure, MoodPanda, iBGStar, Sensaris Senspod, 23andMe, and uBiome. An open coding analytical approach was used to find all the themes related to the research aim.

**Results:**

This study distinguished three types of measurements in health SQ: body structures and functions, body actions and activities, and around the body.

**Conclusions:**

The CDA-SQS classification should be applicable to align health SQ measurement data from people with many different health objectives, health states, and health conditions. CDA-SQS is a critical contribution to a much more consistent way of studying health SQ.

## Introduction

People may use wearable tools acquired in direct-to-consumer market places to undertake various processes or activities related to personal health care (eg, data collection and data analysis) as part of self-selected real-life and Web-based communities [1-3]. This practice can be referred to as health self-quantification (SQ) [4].

The use of wearable tools for health SQ is growing in popularity. Globally, the total number of self-quantifiers is unknown, but data available from Quantified Self Meetup groups from Keyhole websites and about the volume of retail sales and shipments of wearable tools indicate that more people are becoming interested in health self-care every year. The Quantified Self international social movement, founded in 2007, has grown up over a decade. In 2007, there was only one group, in California, United States; by August 2017, there were 238 groups worldwide as indicated by the Quantified Self Meetup website. The number of members of these groups increased from around 14,000 in November 2012 to over 83,000 in August 2017.

In social networks, the Keyhole hashtag analytics tool shows that hashtags such as #QS, #wearables, and #smartwatches have a wide reach (reach refers to the number of unique people who may see the posts from Twitter and Instagram). For example, from April 30, 2016 to May 31, 2016, the hashtags of #QS involved over two million people who were talking about health, fitness, weight, health data, body measures, apps, etc as denoted by the Keyhole’s word cloud.

The volume of retail sales is increasing because people do not want to limit themselves to the functions that are enabled by mobile phone apps alone. They are striving to take advantage of more advanced features enabled by associated wearable tools because these features claim to enable better maintenance or improvement of their health status [5]. In the United States, the retail sales of these tools increased over the first 8 months of 2015 from US $343.5 million to US $754.8 million [6]. Around the world, Apple sold around 13 million units of smart watches in its first year of sale (ie, April 2015), which doubled the sales of the first iPhone [7].

The volume of shipments of wearable tools is also going up. The number of shipments of wearable tools nearly tripled from 2013 to 2015 from 13 million units in 2013 to around 34 million units in 2015. This level of shipments was expected to expand rapidly over the 5 years to 2020 [8,9]. Another study stated that more than 61 million wearable tools would be shipped to mobile phone users in 2017 [10].

Although health SQ may be done as a solitary, private activity, many people choose to share their experiences, reflections, and data. Web-based platforms such as PatientsLikeMe, Genomera, and CureTogether now cater to self-quantifiers. For example, in April 2015, PatientsLikeMe allowed 38,000 members with multiple sclerosis to link and display activity data from their Fitbit trackers [11]. In addition, by 2018, 70% of health care organizations worldwide were predicted to invest in health technologies including apps and wearable tools, according to IDC Health Insights as cited in [12].

Crowdsourcing these data and integrating them with self-quantifiers’ clinical data and history could enable health data analytics research to investigate individuals’ health self-care activities and examine their effectiveness [2,13]. Findings from such research could lead to the development of better-personalized health interventions and ultimately could improve health care in ways that benefit the many people who use a variety of tools to engage in different sorts of health SQ [14,15]. However, these population-scale benefits of health SQ cannot be realized fully without a common language for describing the measurement data generated by the use of the various SQ apps and tools [16]. The lack of a common language or taxonomy in this field prevents the systematic investigations that are needed to provide evidence of sufficient quality to determine whether and how health SQ is a worthwhile health care paradigm, and so to advance the use of SQ in health self-care [17].

The aim of this paper, therefore, was to investigate a significant sample of wearable tools and services to build and test a taxonomy of health SQ measurements. We called this the classification of data and activity in self-quantification systems (CDA-SQS).

## Methods

This section shows the phases of developing CDA-SQS, as follows.

### Phase 1: Building a Preliminary Taxonomy of Health SQ Measurements

**Figure 1:**
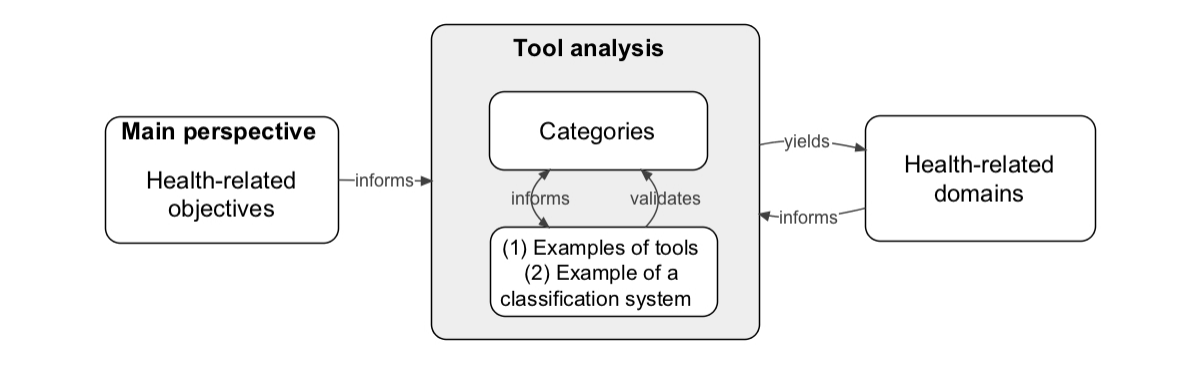
Open coding and comparative assessment used to build a taxonomy of health self-quantification (SQ) measurements.

This phase was concerned with building a preliminary taxonomy of the measurements that self-quantifiers can take using wearable tools to achieve their health objectives (eg, measuring being active by capturing data that you have walked 10,000 steps per day). To achieve this, first, a sample of health SQ tools and services (N=8) was selected to be examined in mid 2012. Then, the analysis of the measurements that could be generated by these tools was carried out by using an open coding technique (Figure 1, see also [18]). The following two subsections explain these steps.

#### Selection of Health SQ Tools

This subsection provides information on the selected tools, selection criteria, and tools’ descriptions.

A sample of health SQ tools and services (N=8) was selected to be examined in this review study: (1) Zeo Sleep Manager, (2) Fitbit Ultra, (3) Fitlinxx Actipressure, (4) MoodPanda, (5) iBGStar, (6) Sensaris Senspod, (7) 23andMe, and (8) uBiome. The sample needed to reflect the different features related to data collection that were available for health self-quantifiers to use and those that Quantified Self groups identified. These included manual and automatic data collection, single-use data collection, and data types, as shown in Table 1.

**Table 1:** Selection criteria of self-quantification (SQ) tools. Table rows do not imply direct correspondences among health SQ tools but rather indicate the presence of a feature in the selected SQ tool or service.

Feature related to data collection	Tool number							
	1	2	3	4	5	6	7	8
Manual data collection			X	X				
Automatic data collection	X	X			X	X		
Single-use data collection							X	X
Data types	Sleep hours and quality	Body movement–related data, for example, steps taken	Blood pressure and pulse	Mood	Blood glucose	Environmental data, for example, ambient humidity	Genome data (single nucleotide polymorphism profile)	Microbiome data

These tools were chosen from a list of forty-two health SQ tools that were being acquired in 2012 to build a SQ research laboratory in the Health and Biomedical Informatics Centre (HaBIC) at the University of Melbourne. The tools acquired for this lab (listed in Multimedia Appendix 1) represented the best selection available in the consumer market place at that time to measure different aspects of health, and so they reflected the existing diversity of health data types that could be generated from using health SQ tools.

The description of the selected tools is based on the following information sources and information-gathering methods:

- Physical inspection of some of the selected tools by researchers. For example, Fitbit Ultra was used by author FM, 23andMe by author KG, and MoodPanda by author MA.
- Information pages that were available on the manufacturer’s or service provider’s website.
- The manufacturer’s technical manuals.
- Articles published in academic journals (eg, [19,20]).
- A number of openly accessible Web-based sources such as the Quantified Self guide; app stores including the Apple Store, Google Play (formerly the Android market), Microsoft Store, and BlackBerry World; and websites such as Vandrico, Wellocracy, and the PatientView directory of health apps.
- Blogs [21].
- Quantified Self “show and tell” meetings; during the PhD study, the researchers were active members of the Melbourne Quantified Self MeetUp group and attended several “show and tell” meetings of this group. During these meetings, Quantified Self members talked about the tools they used for their health SQ. Most of them were coincidentally the same tools that were included in this study (eg,, Zeo Sleep Manager, 23andMe, MoodPanda, and Fitbit Ultra); therefore, based on such users’ accounts, the researchers were able to further enhance the content of the tools review.
- Upon requesting permission to use pictures of SQ tools in publications related to our study, a number of commercial representatives asked to review the researchers’ description of their product. On the basis of that, they offered additional information and suggestions to improve this description, which served as a double check and validation of this part of the study.

It is worth noting that the description of the sampled tools in this paper is based on versions and sources that were available in 2012; because they were selected and reviewed at that time, they are described below in the past tense. Some technical features may have changed by the time of publication. For example, the 2016 version of Fitbit was called Fitbit AltaHR. This system had no base station to be attached to the user’s computer and used Bluetooth to connect the wearable device to the computer or mobile phone. In addition, the Fitbit physical design was changed from a clip that could be attached to clothing into a bracelet that could be worn on the wrist. However, changes in the technical features of the tools that were sampled—and indeed the discontinuation of some tools and the emergence of other new ones—have not altered the value of the method used in this study to build the taxonomy of health SQ measurement. This is because the focus here is not on these tools’ technical features as such but rather on using their description as a first step in the process of typifying the health data types collected by many such tools.

Here are the descriptions of the eight selected tools:

1. Zeo Sleep Manager was a tool for tracking sleep quality, that is, the amount of hours spent in the following four states: light sleep, deep sleep, very deep sleep or dreaming stage, and waking. It comprised a headband that users wore during sleep. The headband measured the brain’s electrical signals and provided a quantitative sleep quality value called the Z-score [19]; on the basis of that, it indicated the state that the person is in. These signals were sent to a bedside-clock device to be stored on an attached secure digital memory card and then analyzed. To see a history of sleep states, the user needed to have Internet access and create an account on mysleep.myzeo.com. The company stopped offering their services in 2013 and has not been accessible since then.
2. Fitbit Ultra was a tool for tracking movement including steps taken, stairs climbed, distance travelled, and movement during sleep; calories burned; and hours of sleep. It consisted of a clip that could be attached to clothing; a base station that could be attached to a Windows personal computer or a Mac, which connected the clip with the paired computer or mobile phone; and the Fitbit app for visualizing, analyzing, and sharing the collected data.
3. Fitlinxx Actipressure was a tool for tracking blood pressure and pulse. It comprised an inflatable rubber cuff and a device that had a large digital panel for displaying blood pressure and pulse readings. The user needed to wear the cuff on the upper arm and press the start button, which caused the cuff to inflate automatically and take measurements. To build and review the history of measurements collected, the user needed to have Internet access and create an account on the ActiHealth website where she or he could access the widget ActivePressure and see their blood pressure and pulse readings. This device connected to the user’s computer wirelessly via an ActiLink personal access point that plugged into the computer’s universal serial bus (USB) port.
4. MoodPanda was a mobile phone–based app for tracking happiness. It allowed users to rate their happiness on a scale from 0 to 10, where 0 was very unhappy and 10 was very happy. Users could also add a brief comment about what was influencing their mood and share their ratings and comments with friends. The app was compatible with different mobile platforms and could be downloaded from app stores such as Apple Store, Google Play (formerly the Android market), Microsoft Store, and BlackBerry World.
5. iBGStar was a blood glucose meter. It consisted of the iBGStar Diabetes Manager app and a blood glucose meter that could be used on its own or attached to an iPhone or iPod touch through the USB port for displaying, tracking, and communicating data of particular interest to people with diabetes. A blood sample was obtained by a lancing tool and placed on the test strip for measuring the blood glucose level. Once the blood glucose meter was attached to the mobile phone, the iBGStar Diabetes Manager app was launched. Then, the readings were automatically logged in the app. If the meter was used alone, the data were saved in the meter’s memory and loaded onto the mobile app upon next connecting to the Internet. The app also allowed the user to email these collected readings to others including health care professionals, or transfer them to computers for storing or analysis.
6. Sensaris Senspod was a device that captured environmental data in real time and sent them via Bluetooth to a paired mobile phone. It could be installed in homes, offices, etc to capture ambient noise, humidity, temperature, and carbon monoxide and nitrogen oxide levels. Senspod was provided with an Android application and access to the Web interface. A user could login through their mobile phone or computer to their account on Sensaris.com to read and share the collected data.
7. 23andMe was a Web-based service for performing a genetic test consisting of single nucleotide polymorphisms profile analysis. Users needed first to order a kit from 23andMe in California, United States through the 23andMe website. The kit consisted of a tube where a sample of the users’ saliva or a cheek swab was placed. Then, the user needed to create a personal account on 23andMe.com and register the tube identity number before sending it to the 23andMe lab in the United States. Within 4 to 6 weeks, the user received reports which described many of their genes and genetic variants that could be associated with risk of diseases and also provided some information about their ancestors [22]. These reports could be used for predicting diseases that might affect the person in the future and hence, could enable a proactive approach to health management [23]. Furthermore, they could be used for designing more personalized treatment of health conditions [22]. Users could also anonymously compare their own results with others who had genomes like theirs, as well as with the latest scientific research findings.
8. uBiome was a service for analyzing the deoxyribonucleic acid of bacteria that exist in the skin, ears, mouth, sinuses, genitals, and gut. Users needed first to order a kit from uBiome in San Francisco, United States through the website. The kit consisted of strips for taking swabs from different body parts (eg, skin, ears, and mouth). The user received a participant identity number upon ordering the kit; this was needed for signing up to create a personal account on uBiome.com. Next, after taking swabs, the user sent the kit back to the uBiome lab. The user then needed to log in to their account to see and analyze the results. A variety of analysis widgets (eg, percentages and distribution frequencies) and related data viewers (eg, bar chart, pie charts, and logbook) could be accessed via the user’s personal account. Users could also anonymously compare their own results with others who had microbiomes like theirs, as well as with the latest scientific research findings.

#### Analysis of Data Types From Health SQ Tools

An inductive content analysis method—also known as open coding—was employed for analyzing measurements, as shown in Figure 1 [24,25]. During open coding, the tools were reviewed and compared iteratively. Specifically, one researcher examined all the data types (eg, sleep and blood glucose) that could be acquired by the eight tools and inductively assigned them to categories. The data types that were similar were grouped together to create a category or a class. If a data type did not fit with the previously created class, a new class would be added. The classes were then grouped into overarching health-related domains. The coding process was repeated until domains and classes reached a stable state, that is, additional tools did not yield new domains or classes. Open coding was conducted by author MA. Authors KG and FM were involved as reviewers in this step to ensure that each theme was representative, as well as to check how similar they were to each other within a group and how different from codes in every other group. On the basis of this process, the arrangement of codes was refined.

The preliminary taxonomy based on analysis of eight tools was revisited (as shown in Figure 1) in relation to the other tools acquired for the HaBIC health SQ lab. Going through this comparative assessment process confirmed that it would not expand the taxonomy significantly if this study were to extend to in-depth examination of all of these tools. In fact, even this wide range of tools did not yet support many types of health-related measurements with which the researchers were familiar (eg, no tools were available that could measure exposure to all air pollution particulates).

Thus, reaching saturation in terms of describing all possible data types in all health-related domains in this phase of the study was not feasible. So there was a need to ground our preliminary taxonomy in relation to an external rigorous classification system, to build the taxonomy as exhaustively as possible. This was done in phase 2.

### Phase 2: Refining the Taxonomy in Relation to Other Classification Systems

The second phase, in mid 2013, sought to refine the preliminary taxonomy (as shown in Figure 1) through comparative assessment with an already established system for classifying health.

For this purpose, we looked for a classification system that met the following criteria: developed by an internationally recognized health organization, comprehensive, health and functioning must be the basic organizing concept, able to fit within an external framework to contribute to a more consistent way of studying health SQ, and able to accommodate conventional and unconventional observations of potential influences on an individual’s health. Three international classification systems were identified and considered: the World Health Organization’s International Classification of Functioning, Disability, and Health (WHO-ICF) [26]; the European Directory of Health Apps [27]; and Happtique [28]. Comparative descriptions of each one are provided in Multimedia Appendix 2.

WHO-ICF was selected because it met all the stated criteria, and it provided the most comprehensive list of health measurements—it had more than 1400 categories [29], whereas the European Directory of Health Apps and Happtique, respectively, had around 60 and 300 categories. Health and functioning were its basic organizing concept [26,30]. It could be coupled with external frameworks for describing health concepts [26,30]. It defined the individual’s health function or disability in relation to what they can do or cannot do as a dynamic interaction between the individual, their personal factors (eg, age and gender), and their environmental factors (eg, ambient weather temperature and humidity and use of communication technologies such as mobile phones) [26,31,32].

At the end of phase 2, the taxonomy was expanded through this comparison and refinement of categories; however, it remained theoretical. So a third phase of the study was carried out to test it in relation to real-world health SQ.

### Phase 3: Validating the Taxonomy in Relation to Self-Quantifiers’ Practices

To validate the taxonomy, it was examined in relation to self-reported activities of health self-quantifiers. This information was derived from the results of an international Web-based survey that we conducted in 2014 to explore many aspects of people’s use of a wide range of health SQ tools and services. More details about the broad aim, design, and results of this survey is available in [33].

Pertinent to validating the theoretical taxonomy of health SQ measurements, among the survey’s 67 questions were multiple-choice questions, as well as open-ended questions to elicit information about the names and the number of tools used by an individual and the kinds of data that the individual collected using these tools. The survey results enabled us to revisit our categories in relation to responses about the use of 130 different SQ tools. These responses were provided by 103 self-quantifiers who varied in age, gender, health status, and motivation.

For the purpose of validation, the researchers qualitatively analyzed these responses using deductive thematic coding based on the theoretical taxonomy. Once again, initial coding was conducted by author MA and reviewed by authors KG and FM in this step. We observed whether and to what extent it was possible to account for the tools used and the data types generated from these tools within corresponding health domains and categories in the taxonomy.

## Results

**Figure 2:**
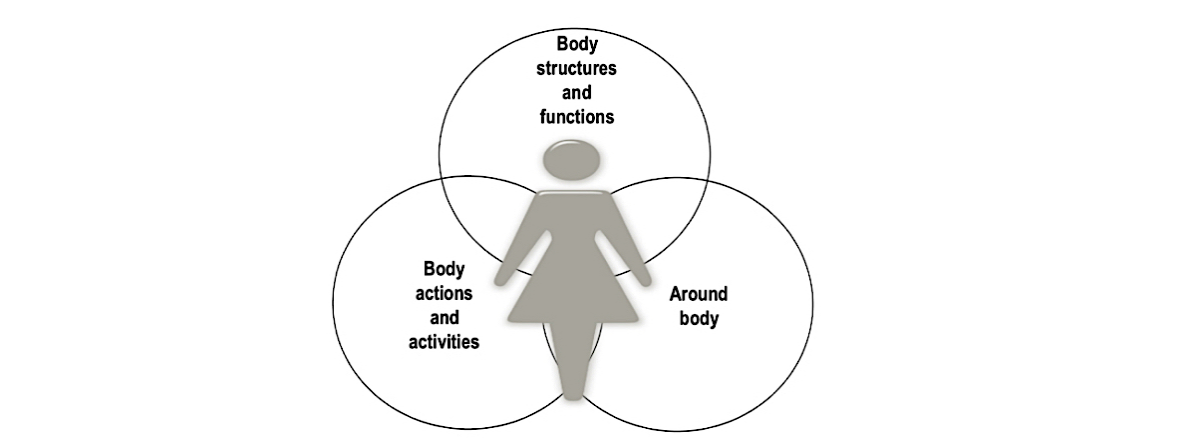
The interactive model of classification of data and activity in self-quantification systems (CDA-SQS).

Phase 1 led to the development of a preliminary taxonomy that we called CDA-SQS, as shown in Table 2. Phase 2 led to the development of a model of the measurements in health SQ, as shown in Figure 2, as well as to the improvement of the taxonomy in terms of increasing the number of health measurement categories that it could account for, as illustrated in Table 3. Phase 3 led to refinement to the CDA-SQS model and showed the applicability of CDA-SQS for characterizing measurements in health SQ. The following paragraphs explain the results from these three phases in more detail.

**Table 2:** The health domains and corresponding categories of the selected self-quantification (SQ) tools. The numbers from 1 to 8 indicate the SQ tool or service we analyzed. “X” indicates the SQ tool or service’s functionality to capture this type of measurement.

Domain name	CDA-SQS^a^		SQS							
Health domain	Category	Subcategory	1	2	3	4	5	6	7	8
Body functions	Mental functions	Sleep	X	X						
	Mental functions	Emotions				X				
	Cardiovascular system	Blood pressure			X					
	Endocrine system	Blood glucose					X			
Body structures	Cell structure	Genes, deoxyribonucleic acid, etc							X	
	Microbial structure in skin, gut, etc	Names, number, types, etc								X
Body actions and activities	Mobility	Walking		X						
Around the body	Natural environment	Climate or weather						X		

^a^CDA-SQS: Classification of data and activity in self-quantification systems.

**Table 3:** A two-level summary of the classification of data and activity in self-quantification systems (CDA-SQS). Table rows do not imply direct correspondences among health self-quantification measurements but rather indicate the wide variety of combinations that a self-quantifier may choose to explore.

Body structures	Body functions	Body actions and activities	Around the body
Cell structure Microbial structure in skin, ears, mouth, sinuses, genitals, and gut Structure of brain The eyes, ears, nose, mouth, pharynx, and larynx Structure of cardiovascular system Structure of immune system Structure of respiratory system Structure of digestive system Structure of endocrine glands Structure of urinary system Structure of reproductive system and pelvic floor Structure of musculature Structure of spinal cord Structure of skin, hair, and nails	Mental functions Sensory functions Sensation of pain Voice and speech Functions of the cardiovascular system Functions of the hematological system Functions of the immune system Functions of the respiratory system Functions of the digestive system Functions of the metabolic system Functions of the endocrine system Genitourinary functions Reproductive functions Neuromusculoskeletal and movement-related functions Functions of skin, hair, and nails	Learning and applying knowledge General tasks and demands Communication Mobility Self-care Domestic life Recreation and leisure Religion and spirituality	Natural and built environment Relationships and attitudes Community, social, and civic life Major life areas Products and technologies Time

### Results From Phase One

Table 2 presents the results from the open coding that was conducted in the first phase. It shows the categories of what people would be measuring, if they were using the SQ tools that we examined, to understand their body and health status. These tools were grouped by the data types into (1) inside the human body, in which data were related to bodily functions (N=4) and structures (N=2); (2) on the body, which was concerned with bodily actions and activities (N=1); and (3) around the body (N=1), which pertained to bodily surrounds including environmental aspects that might affect the individual’s health. Therefore, CDA-SQS categorizes measurements into three main health-related domains: body structures and functions, body actions and activities, and around the body.

### Results From Phase Two

Table 3 illustrates the CDA-SQS domains and related categories; this represents the result from the second phase. The CDA-SQS presents the three domains and their related categories and subcategories in a hierarchical way. The following paragraphs describe CDA-SQS through comparing and contrasting it with WHO-ICF. Multimedia Appendix 3 displays a detailed description of the taxonomy developed from this study.

The WHO-ICF taxonomy categorizes measurements into three main health-related domains: body functions and structure, activity and participation, and environmental factors (eg, the natural and built environment). In CDA-SQS, the domain “in body” is more or less similar to the corresponding one in WHO-ICF because they both describe aspects related to functioning at the level of the body [34]. However, CDA-SQS added new categories. For example, the class “microbial structure in skin, ears, mouth, sinuses, genitals, and gut” was added to the category “body structures.”

In WHO-ICF, the second domain is about aspects of functioning at the level of the individual (ie, one’s activities) and at the social level (ie, one’s participation in society), whereas environmental factors are conceived as facilitators or barriers to the person’s functioning at these levels [34]. However, the distinction between functioning at the level of the individual and functioning at the social level is not clear; it has proved problematic to operationalize, and it has been argued that categories related to the activity and participation domain should belong to two different domains [31]. This was done in CDA-SQS as follows.

In CDA-SQS, activities now belong to the “body actions and activities” domain because they are related to functioning at the level of the individual, whereas participation and environmental factors belong to the “around the body” domain because they describe surrounding aspects that may affect the individual’s health. Consequently, the categories related to the domains “activity and participation” and “environmental factors” in WHO-ICF were rearranged to fit the purpose of CDA-SQS. For example, categories such as “mobility,” “general tasks and demands,” “communication,” “learning and applying knowledge,” and “self-care” were placed under the body actions and activities domain, whereas “major life areas” and “community, social, and civic life” were placed under the “around the body” domain.

Some categories in WHO-ICF were complicated, such as “functions of the cardiovascular, hematological, immune, and respiratory systems.” Such categories were rendered into a number of corresponding categories; in this instance, it was rendered into four categories: functions of the cardiovascular system, functions of the hematological system, functions of the immune system, and functions of the respiratory system.

In contrast, some WHO-ICF categories such as “interpersonal interactions and relationships” and “attitude” created distinctions that were not useful. These were combined into one CDA-SQS category, in this case called “relationships and attitudes.” We argue that for the purpose of health SQ, measuring interactions and relationships is more than just counting the number of them. What most self-quantifiers are interested to track and explore are the attitudes that they hold toward these social relations, the attitudes they experience within them, and how these attitudes affect their personal well-being.

Overall, CDA-SQS is consistent with WHO-ICF in major respects. It structures domains into categories and subcategories, as WHO-ICF does. It can fit within external frameworks to capture contextual data about self-quantifiers for studying their health status, as WHO-ICF can. For example, CDA-SQS can be integrated with a framework called minimal information about SQ experiment [35,36]; hence, data about a self-quantifier’s personal factors (eg, age, gender, and education level) and more can be captured.

### Results From Phase Three

Our survey showed that to better understand the health status, individual self-quantifiers tend to collect multiple types of health data, which may be in different health domains, for example, the connection between sleep patterns and weather patterns. Therefore, the CDA-SQS model is illustrated as three intersecting circles, as depicted in Figure 2. This refinement is in agreement with WHO-ICF; both systems conceive of health domains and categories as interactive and an individual’s health as the product of interaction among components.

The CDA-SQS classification was also found to be applicable to all of different kinds of health measurements (N=130) that survey participants reported taking with SQ tools. Every type of measurement could be fitted into one of these health-related domains: “body structures and functions” (N=53), “body actions and activities” (N=39), and “around the body” (N=16). Each of these domains has a hierarchy of categories and subcategories. Multimedia Appendix 4 shows the classification of a subset of kinds of measurements taken using SQ tools (N=108) within these three domains. The remaining 22 tools were found to capture a range of health aspects that belong to more than one health domain denoted by the CDA-SQS; for the sake of clarity, they were listed in Multimedia Appendix 4 but not presented in the appendix table.

## Discussion

CDA-SQS provides a common language for describing measurements generated from SQ tools. This was a largely untapped area in prior literature: our systematic literature reviews [4,37] revealed limited attempts from researchers to classify SQ data in a comprehensive or systematic manner. One study collected 209 users’ reviews from the Quantified Self website [38] and, using inductive content analysis, classified health SQ data into five categories: body state (eg, physical and physiological), psychological state and traits, activities (eg, exercise, eating, and sleeping), social interactions, and environmental and property states. However, we found this classification inadequate to account for all of the health-related aspects in each category that our research uncovered. CDA-SQS provides a comprehensive description of measurements generated from SQ tools in comparison with the classification developed by [38].

CDA-SQS is also more versatile than the non-peer reviewed classifications we identified. For example, CDA-SQS identifies more health categories than the myhealthapps directory 2015-2016 [39]. Although that directory was built by inductively analyzing 300 health-related apps recommended by consumers for health self-care, it classifies these apps into only two main domains (ie, disability and health) and 21 corresponding categories. In contrast, CDA-SQS was developed initially by inductively analyzing eight SQ tools and nominating three main health domains; however, the subsequent phases in its development process led to generating 43 categories under these three domains, further broken down into 124 subcategories.

In CDA-SQS, health and functioning is the basic organizing concept; this is in line with the current orientation in delivering health care [30,40]. Furthermore, it can be used to augment other analytical tools that are necessary to investigate health SQ rigorously: it fits with our SQS taxonomy for classifying tools and services [41]. It can be integrated with our theoretical framework for dissecting health SQ activity [37]. It is compatible with models such as our Personal Health Information Self-Quantification Systems [33] and our health SQ chain value model [4]. In this way, CDA-SQS is a critical contribution to a more holistic and consistent way of studying health SQ; SQ data can be put into an individual’s contexts of use—in relation to the measurements denoted by CDA-SQS, to the tools and services described by SQS taxonomy, to the health self-management activities illustrated by our models, and to the personal demographics and the community connections shown by the health SQ activity framework. Furthermore, CDA-SQS can account for relatively conventional observations (eg, cardiovascular function) and also less conventional observations (eg, gut microbes and environmental exposures) about an individual’s health. This in turn may help in studying the interplay of these aspects of health and ultimately in developing more personalized health interventions [42].

This study has the strength that arises from collaborative use of mixed qualitative methods within a research team. Critically, it used both inductive and deductive logic to examine the health-related measurements generated from the use of SQ tools and services. This led not only to developing a classification schema but also to establishing a conceptual framework for describing measurements in health SQ. However, this study has some limitations. In the inductive content analysis, complete interrater reliability analysis could not be conducted because this study was conducted as part of author MA’s PhD student, and so MA had to be the only coder in phases 1 and 3 [43]. As explained in the Methods section, the other authors were MA’s PhD supervisors and so could provide oversight and critique but not full reliability testing. Thus, a follow-up study to test CDA-SQS performance on a wide range of health SQS in laboratory or field trials would shed more light on the results of this study. Other methods too could be used to test the validity of phase 2, for example, eliciting review and feedback from health care professionals and from more members of the Quantified Self community.

This study has articulated a formal methodological approach for describing and distinguishing the different types of measurements in health SQ. The resulting CDA-SQS classification is applicable to many different types of scientific studies that involve SQ measurements taken by people regardless of their health objective, health status, or health condition, and regardless of the tool or service they use for this purpose.

Establishing protocols that can be used to work with health self-quantifiers’ measurements is a necessary biomedical informatics foundation for reporting experimental results in this area. CDA-SQS has shown its usefulness in establishing a common format and reporting guideline for health SQ studies [35,36]. These informatics tools may serve as the basis for improving the design of research databases, including public repositories, where users of health SQS choose to contribute their data. Our future studies aim to test CDA-SQS applicability for such purposes.

## Appendix

Multimedia Appendix 1 List of SQ tools available in the HaBIC SQ lab.

Multimedia Appendix 2 Description of and comparison between three classification systems.

Multimedia Appendix 3 Classification of data and activity in self-quantification systems.

Multimedia Appendix 4 Applicability of CDA-SQS.

## Abbreviations

CDA-SQS: classification of data and activity in self-quantification systems
HaBIC: Health and Biomedical Informatics Centre
SQ: self-quantification
WHO-ICF: World Health Organization’s International Classification of Functioning, Disability, and Health
