# Supplementary material for: Development and Validation of a Taxonomy for Characterizing Measurements in Health Self-Quantification
Source: J Med Internet Res. 2017 Nov 3;19(11):e378. doi: 10.2196/jmir.6903 (PMC5694028; doi:10.2196/jmir.6903)
Supplement: Multimedia Appendix 1 [file jmir_v19i11e378_app1.pdf]

Appendix 1: List of SQ tools available at self-quantification Lab

The table below presents a list of all SQ devices available at Health and Biomedical Informatics Centre at the University of Melbourne self-quantification Lab since 2013.

Data type	SQ Device
**Physical Activity**	Body Media FIT
	Body Media FIT Wearable Display - BS100735
	BodyMedia FIT + Armband + Display BS 100734
	BodyMedia FIT Display Wristband Accessory - BS100179
	Fitbit Flex
	Fitbit Zip
	Fitbit Zip Wireless Activity Tracker
	iWatch
	Jawbone Up
	Misfit Shine
	Phyode W/Me Tracker
	Polar RCXBike
	Samsung Galaxy Gear Smartwatch
	Sense U Activity
	Suunto Ambit2GPS+Hearth Rate Monitor Watch
	Withings Pulse Wireless
**Weight**	Accumeasure Fitness 3000 Body Fat Tester
	Fitbit Aria Wi-Fi Smart Scale - FB201B
	Withings Smart Body Analyzer
**Sleep**	Fitbit One Wireless Activity and Sleep Tracker - FB
	Lark Pro Sleep Monitor Silent Alarm Clock and Personal Sleep Coach
	Renew Sleep Clock - PG 754
	Sleep Tracker
	Zeo Mobile Sleep Management System ZEOMBL1 + docking station
**Blood**	Beurer Mobile ECG Device - ME80
	Beurer Upper Arm Digital Blood Pressure Monitor - BM58
	Garmin Forerunner Heart Rate Monitor
	IBGStar Blood Glucose Meter Kit
	iHealth Wireless Blood Pressure Monitor Upper Arm Cuff - IH-BP5
	iHealth Wireless Pulse Oximeter - IH-PO3
	Tinke Heart Rate Monitor - 2414458
	Viiiiva Dual Heart rate Monitor
	Withings Blood Pressure Monitor
**Environment**	Tempe Wireless Temperature
	Lapka Personal Environment Monitor for iPhone
**Respiratory**	Airsonea
**Brain**	Mind Reflector
	Mindwave Mobile
**Posture**	LUMO - Back Posture Sensor
	WiTouch Pro Tens - Pain Management
**Microbial structure in human body**	ubiome
**Cell structure**	23andMe
