# Supplementary material for: Development and Validation of a Taxonomy for Characterizing Measurements in Health Self-Quantification
Source: J Med Internet Res. 2017 Nov 3;19(11):e378. doi: 10.2196/jmir.6903 (PMC5694028; doi:10.2196/jmir.6903)
Supplement: Multimedia Appendix 2 [file jmir_v19i11e378_app2.pdf]

Appendix 2: Description of and comparison between three classification systems

The researcher MA reviewed and compared three classification systems: International Classification of Functioning, disability and health (WHO-ICF), the European Directory of Health Apps, and Happtique in order to decide which one can be employed for refining and extending the CDA-SQS taxonomy. The researcher looked for a classification system which met the following criteria: 1) developed by recognised international health organisations; 2) comprehensive; 3) health and functioning must be the basic organising concept; 4) can fit within an external framework in order to be integrated with our health self-quantification activity framework [14]; and 5) can account for conventional and unconventional observations of potential influences on the individuals for defining health.

International classification of functioning (ICF) was developed by the World Health Organisation (WHO) and published in 2001 [19]. ICF was named so because its focus was on health, rather than on disability [15,19]. WHO-ICF is composed of a comprehensive taxonomy for describing measurements in health, and a theoretical framework underpinning this taxonomy. ICF framework defines the health or person's functioning as a dynamic interaction between health conditions and environmental and personal factors [31]. On the other hand, WHO-ICF taxonomy divides measurements in health into three health related domains: body functions and structure (e.g., mental functions, functions of the cardiovascular systems, structure of respiratory system); activity and participation (e.g., mobility in term of transferring from one place to another); and environmental factors (e.g., natural and built environment). A brief core set of the WHO-ICF taxonomy is presented at: http://apps.who.int/classifications/icfbrowser.

The European Directory of Health Apps 2012-2013 was developed by PatientView (http://www.patient-view.com/bull-who-we-are.html) and published in 2012. It involves a simpler classification system in compared with WHO-ICF, and a directory of apps. In this review, the researcher was interested in the classification system. The classification system assigned about 200 health-related apps into 62 categories based on their health specialities (e.g., cancer, Alzheimer disease, etc.). Thus, health related disease was the basic organising concept in this classification system. In contrast, the newer edition of the directory is organised around the concepts of health and disability. For further information about the directory, please check the following link: http://www.patient-view.com/-bull-directories.html.

Happtique provides a simpler classification system in compared with WHO-ICF. It was founded in 2010 by the venture arm of the Greater New York Hospital Association. The company offers a platform for the certification, prescribing, and curation of mobile health apps. In this review, the researchers were interested in the hApp Catalogue. The hApp Catalogue contains a list of apps that are indexed into more than 300 categories based on health conditions [17]. Thus, health related diseases were the basic organising concept in this classification system. In 2012, the hApp Catalogue was publicly available and could be accessed by anyone online at: happtique.com. However, this is not the case anymore. In early 2013, the company suspended the program, and in 2014, it was acquired by a digital health company called SocialWellth (http://socialwellth.com). There have not been new updates regarding the hApp Catalogue from SocialWellth.

The following table summarises the comparison between these classification systems.

Criterion No.	Criterion Description	WHO-ICF	European Directory of Health Apps	Happtique
1	**Founding health organisations**	World Health Organisation (WHO).	PatientView.	Greater New York Hospital Association.
2	**Comprehensiveness (approximate number of categories)**	1,400 categories.	62 categories.	300 categories.
3	**Organising concept in the classification system**	Categorising measurements in the context of the person health functioning and restrictions.	Categorising measurements based on health specialities (e.g., cancer, Alzheimer disease, etc.).	Categorising measurements in the context of the person health conditions using the vocabulary of healthcare professionals.
4	**Fitting within an external framework**	Yes.	Not stated.	Not stated.
5	**Defining the individuals' health**	Defines the individual's health as a dynamic interaction between the individuals, their personal factors, and the environmental factors.	Does not account for factors of potential influences on the individual's health.	Does not account for factors of potential influences on the individual's health.
