# Supplementary material for: Development and Validation of a Taxonomy for Characterizing Measurements in Health Self-Quantification
Source: J Med Internet Res. 2017 Nov 3;19(11):e378. doi: 10.2196/jmir.6903 (PMC5694028; doi:10.2196/jmir.6903)
Supplement: Multimedia Appendix 3 [file jmir_v19i11e378_app3.pdf]

Appendix 3: Classification of Data and Activity in Self-Quantification Systems

BODY STRUCTURES AND FUNCTIONS	
**Domain**	**Category**
**Body functions class**	
MENTAL FUNCTIONS	Appetite<br>Attention e.g. sustaining attention, shifting attention, dividing attention, sharing attention; concentration; distractibility<br>Calculation e.g. manipulation of mathematical symbols and processes<br>Consciousness<br>Emotions e.g. affect; sadness, happiness, love, fear, anger, hate, tension, anxiety, joy, sorrow; lability of emotion; flattening of affect<br>Intellectual growth, mental retardation, dementia<br>Memory e.g. remembering; recalling and learning, amnesia<br>Motivation<br>Perception e.g. recognising of auditory, visual, olfactory, gustatory, tactile and visuospatial perception, such as a hallucination or illusion<br>Psychomotor factors e.g. excitement and agitation, posturing, catatonia, negativism, ambitendency, echopraxia and echolalia<br>Psychosocial functions e.g. autism<br>Sleep<br>Temperament and personality e.g. extraversion, introversion, agreeableness, conscientiousness, psychic and emotional stability, and openness to experience; optimism; confidence; trustworthiness<br>Thought e.g. pressure of thought, flight of ideas, thought block, incoherence of thought, tangentiality, circumstantiality, delusions, obsessions and compulsions, productivity
SENSORY FUNCTIONS	Senses e.g. seeing, hearing, tasting, smell, touch
SENSATION OF PAIN	Pain in back<br>Pain in chest<br>Pain in head and neck<br>Pain in joints<br>Pain in lower limb<br>Pain in stomach or abdomen<br>Pain in upper limb
VOICE AND SPEECH	Voice e.g. pitch, loudness, aphonia, dysphonia, hoarseness, hypernasality and hyponasality
FUNCTIONS OF THE CARDIOVASCULAR SYSTEM	Heart functions e.g., heart rate, rhythm and output<br>Blood vessel functions e.g., functions of arteries, capillaries and veins<br>Blood pressure functions
FUNCTIONS OF THE HAEMATOLOGICAL SYSTEM	Functions of blood production include impairments such as in anaemia, haemophilia<br>Oxygen-carrying functions of blood<br>Metabolite-carrying functions of blood<br>Clotting

FUNCTIONS OF THE IMMUNE SYSTEM	Immune response<br>Hypersensitivity reactions
FUNCTIONS OF THE RESPIRATORY SYSTEM	Respiration functions includes respiration rate, rhythm and depth; impairments such as apnoea, hyperventilation, irregular respiration, etc.<br>Respiratory muscle functions<br>Exercise tolerance functions includes: functions of physical endurance, aerobic capacity, stamina and fatigability
DIGESTIVE SYSTEM	Defecation e.g. faecal consistency, frequency of defecation, diarrhoea<br>Digestive system e.g. dysphagia, vomiting, amount of salivation
METABOLIC SYSTEM	Body temperature<br>Dehydration and rehydration include water intake<br>Metabolism of carbohydrate, protein and fat<br>Mineral balance
ENDOCRINE SYSTEM	Hormonal levels in the body, including cyclical changes, blood sugar (insulin level)
GENITOURINARY FUNCTIONS	Collection of urine e.g. frequency of urination, urinary continence and structure of urine e.g. colour, smell, amount
REPRODUCTIVE FUNCTIONS	Menstruation including regularity of menstruation and discharge of menstrual fluids<br>Procreation e.g. fertility, pregnancy<br>Sexual act e.g. orgasmic and resolution phase
NEUROMUSCULOSKELETAL AND MOVEMENT-RELATED FUNCTIONS	Mobility of bone e.g. the scapula, pelvis, carpal and tarsal bones.<br>Mobility of joint e.g. shoulder, elbow, wrist, hip, knee, ankle, small joints of hands and feet<br>Muscle e.g. the power of specific muscles and muscle groups, muscles of one limb, one side of the body, the lower half of the body, all limbs, the trunk and the body as a whole
FUNCTIONS OF SKIN, HAIR, NAILS	Function of skin glands, hair, nails
**Body structures class**	
CELL STRUCTURE	Genes, DNA, etc.
MICROBIAL STRUCTURE IN SKIN, EARS, MOUTH, SINUSES, GENITALS AND GUT	What microbiomes, their number, types, etc.
STRUCTURE OF BRAIN	Forebrain, midbrain, and hindbrain, etc.
THE EYES, EARS, NOSE, MOUTH, PHARYNX, LARYNX	All related structures
STRUCTURE OF CARDIOVASCULAR SYSTEM	Heart, arteriesm, veins, capillaries, etc.
STRUCTURE OF IMMUNE SYSTEM	Lymphatic vessels, lymphatic nodes, thymus, spleen, bone marrow, etc.
STRUCTURE OF RESPIRATORY SYSTEM	Trachea, lungs, thoracic cage, muscles of respiration, etc.
STRUCTURE OF DIGESTIVE SYSTEM	Salivary glands, oesophagus, stomach, intestine, intestine, pancreas, liver, etc.

STRUCTURE OF ENDOCRINE GLANDS	Pituitary gland, thyroid gland, parathyroid gland, adrenal gland, etc.
STRUCTURE OF URINARY SYSTEM	Kidney, ureters, urinary bladder, urethra, etc.
STRUCTURE OF REPRODUCTIVE SYSTEM AND PELVIC FLOOR	Ovaries, uterus, breast and nipple, vagina and external genitalia, testes, penis, prostate, etc.
STRUCTURE OF MUSCULATURE	Head and neck region, shoulder region, upper extremity, pelvic region, lower extremity, trunk, etc.
STRUCTURE OF SKIN, HAIR, NAILS	All related structures like the epidermis and dermis, etc.
STRUCTURE OF SPINAL CORD	Spine posture, etc.
**BODY ACTIONS/ACTIVITIES**	
LEARNING AND APPLYING KNOWLEDGE	Learning includes: copying, rehearsing, learning to read, learning to write, learning to calculate, acquiring skills, solving problems, and making decisions.
GENERAL TASKS AND DEMANDS	Undertaking a single task<br>Undertaking multiple tasks<br>Carrying out daily routine<br>Handling responsibilities, stress, crisis, and other psychological demands
COMMUNICATION	Communicating with - receiving - producing - nonverbal messages includes: body gestures, signs and symbols, drawings and photographs<br>Communicating with - receiving - producing - spoken messages e.g. phone calls, videos, using YouTube<br>Communicating with - receiving - producing - written messages e.g. emails, text messages, blogs, social networks<br>Communicating with - receiving - producing - formal sign language messages
MOBILITY	Changing and maintaining basic body inclusion: changing body position from lying down, from squatting or kneeling, from sitting or standing, bending and shifting the body's centre of gravity<br>Climbing includes distances of climbing<br>Crawling includes distances of crawling<br>Hand and arm use e.g. picking up, grasping, manipulating and releasing pulling or pushing objects; reaching; turning or twisting the hands or arms; throwing; catching<br>Jogging includes distances of jogging<br>Jumping and skipping<br>Lifting, carrying, and moving objects e.g. raising up an object or taking something from one place to another, such as when lifting a cup, carrying a child from one room to another, kicking a ball or pushing pedals on a bicycle<br>Moving around in different locations e.g. in a house, within a building, or down the street of a town.<br>Running and scampering includes distances of running<br>Swimming includes distances of swimming<br>Moving around using transportation such as being

	driven in a car or on a bus, rickshaw, jitney, animal-powered vehicle, or private or public taxi, bus, train, tram, subway, boat or aircraft.<br>Walking includes distances of walking, walking on different surfaces such as on grass, gravel or ice and snow, or walking aboard a ship, train or other vehicle.
SELF-CARE	Consumption of drug or medicine<br>Diet<br>Dressing<br>Drinking includes drinking water<br>Eating<br>Washing and drying oneself includes: washing body parts, washing whole body<br>Toileting includes: regulating urination, regulating defecation, menstrual care<br>Weight maintenance
DOMESTIC LIFE	Caring for household objects includes: making and repairing clothes, maintaining dwelling and furnishings, maintaining domestic appliances, maintaining vehicles, maintaining assistive devices, taking care of plants, indoors and outdoors, taking care of animals<br>Household tasks includes: preparing meals, doing housework<br>Acquisition of goods and services includes: shopping and gathering daily necessities<br>Acquiring a place to live includes: buying, renting, furnishing a place to live<br>Assisting others with self-care, movement, communication, interpersonal relations, nutrition, health maintenance
RECREATION AND LEISURE	Engaging in any form of play, sports, arts and culture, crafts, hobbies, or relaxation time e.g. going to art galleries, museums, cinemas or theatres
RELIGION AND SPIRITUALITY	Engaging in religious or spiritual activities such as is involved in attending a church, temple, mosque or synagogue, praying or chanting for a religious purpose, and spiritual contemplation
**AROUND BODY**	
NATURAL AND BUILT ENVIRONMENT	Climate/weather e.g. temperature, humidity, atmospheric pressure, precipitation, wind and seasonal variations<br>Population density<br>Place e.g. Locate the places you want to go to and directions.
RELATIONSHIPS AND ATTITUDES	Domesticated animals e.g. pets (dogs, cats, birds, fish, etc.) and animals for personal mobility and transportation.<br>Extended family, acquaintances, peers, colleagues, neighbours and community members<br>Friends<br>Health professionals e.g. doctors, nurses, physiotherapists, occupational therapists, speech therapists, audiologists, orthotist-prosthetists, medical social workers.<br>Immediate family e.g. spouses, partners, parents,

	siblings, children, foster parents, adoptive parents and grandparents.<br>Personal care providers and personal assistants e.g. providers of support for home-making and maintenance, personal assistants, transport assistants, paid help, nannies and others who function as primary caregivers.
COMMUNITY, SOCIAL AND CIVIC LIFE	Socialising and volunteering includes: engaging in charitable organizations, service clubs or professional social organizations, informal and formal associations; ceremonies
MAJOR LIFE AREAS	Economic self-sufficiency to ensure economic security for present and future needs<br>Education<br>Work and employment e.g. numbers of hours at work<br>Political life and citizenship, human rights
PRODUCTS AND TECHNOLOGIES	Use of assets e.g. money, goods, property<br>Use of products and technologies in activities of moving inside and outside buildings e.g. buses, cars, vans<br>Use of products and technology for communication e.g. television and video equipment, telephone devices, sound transmission systems and face-to-face communication devices, use of computers/smartphones e.g. journaling (blogs and status updates), browsing, tracking, online shopping, etc. It also includes computer/ smartphone usage<br>Use of products and technologies for education e.g. books, manuals, educational toys, computer hardware or software<br>Use of products and technologies for employment to facilitate work activities e.g. scanners, remote control systems, voice-controlled systems and timer switches<br>Use of products and technologies for the conduct and enhancement of cultural, recreational and sporting activities, such as toys, skis, tennis balls and musical instruments<br>Use of products and technologies for the practice of religion or spirituality e.g. crucifixes, menorahs and prayer mats<br>Use of products and technologies that assist people in daily living, such as prosthetic and orthotic devices, neural prostheses<br>Use of products and technologies that assist people in environmental control units aimed at facilitating individuals' control over their indoor setting (scanners, remote control systems, voice-controlled systems, timer switches)
TIME	Natural, regular or predictable temporal change. Inclusions: day/night and lunar cycles
