# Supplementary material for: Development and Validation of a Taxonomy for Characterizing Measurements in Health Self-Quantification
Source: J Med Internet Res. 2017 Nov 3;19(11):e378. doi: 10.2196/jmir.6903 (PMC5694028; doi:10.2196/jmir.6903)
Supplement: Multimedia Appendix 4 [file jmir_v19i11e378_app4.pdf]

Appendix 4: Applicability of CDA-SQS

**Domain**		
**Category**	**Sub-category**	**Primary SQ tools/services**
**Body functions**		
Mental functions	Attention including: concentration; sharing attention, etc.	Melon
	Sleep	Lark<br>SleepCycle,<br>SleepTracker<br>WakeMate<br>Zeo Sleep Manager
	Emotions	Emotiv Insight<br>Happiness<br>MercuryApp<br>Moodjam<br>MoodPanda<br>MoodScope<br>TrackYourHappiness
	Thought including: flight of ideas, thought block, productivity, meditation, etc.	Muse<br>Quantified Mind<br>RescueTime
Functions of the cardiovascular system	Blood pressure	A&D Blood Pressure Monitor<br>Actipressure<br>Blipcare<br>Blood Pressure Companion<br>iHealth
	Heart functions e.g., heart rate, rhythm and output	Adidas miCoach<br>Alivecor<br>Basis<br>Cardiio<br>EmWave2<br>Garmin Heart Rate Monitor<br>Ithlete<br>Mio<br>Myithlete<br>NeuroSky<br>Polar<br>Tinke<br>Wahoo Heart Rate Monitor
Endocrine system functions	Hormonal levels in the body (Blood glucose)	CareLogger<br>Dexcom<br>Glooko<br>iBGStar
Reproductive functions	Menstruation including regularity of menstruation and discharge of menstrual fluids	Clue for tracking cycles<br>Free Menstrual Calendar<br>My Cycles Period and Ovulation Tracker<br>MyFertilityMD
Sensory functions	Senses e.g. seeing, hearing, tasting, smell, touch	EyeNetra
Sensation of pain	Pain in back, chest, head, etc.	Symple<br>TrackNShare
**Body structures**		
Cell structure	Genes, DNA, RNA, etc.	23andMe<br>Eclipse for self breast exam

		InsideTracker<br>Talking20<br>WellnessFX
Microbial structure in skin, gut, etc.	Names, number, types, etc.	uBiome
Neuromusculoskeletal and movement-related functions	Mobility of bone; joints; muscles	LumoBack<br>MYO
**Body actions and activities**		
Mobility	Climbing, crawling, jogging, running, swimming, walking, etc.	Amiigo<br>Bodymedia<br>Endomondo<br>Fitbit One<br>Fitbit Ultra<br>Fitbit Zip<br>Fitocracy<br>Garmin watch<br>iSki Tracker<br>Jawbone Up<br>Misfit Shine<br>Moves<br>MyFitnessPal<br>Nike Fuelband<br>Nike Hyperdunk<br>Nike+ Running<br>Omron<br>Pebble<br>RunKeeper<br>Runtastic<br>Strava<br>Wahoo Fitness<br>Withings
Self-care	Weight maintenance	Fitbit Aria scale<br>Fitorbit<br>Lose It!<br>Retrofit<br>Withings scale
	Diet	80 Bites<br>Dan's Plan<br>Hapilabs fork<br>mySymptoms<br>NutriMirror
	Consumption of drug or medicine	Medisafe Project<br>Medminder<br>Spectracell<br>Vitality Glowcaps
	Washing and drying oneself includes: washing body parts, washing whole body	Toothbrush app
Religion and spirituality	Engaging in religious or spiritual activities	Equanimity Meditation Timer & Tracker
**Around body**		
Natural and built environment	Climate/weather e.g. temperature, humidity, atmospheric pressure, precipitation, wind and seasonal variations, etc.	Lapka<br>Nest<br>Netatmo<br>Sensaris Senspod
	Place e.g. Locate the places you want to go to and	Chronos<br>Garmin GPS

	directions.	Geonaute Keymaze<br>Placeme<br>WorkInField
Major life areas	Economic self-sufficiency to ensure economic security for present and future needs	Mint<br>Slice<br>TheBirdy<br>TOSHL finance
Products and technologies	Use of products and technologies in activities of moving inside and outside buildings e.g. buses, cars, vans, airplanes, etc.	TripIt flights
Relationships and attitudes	Personal care providers and personal assistants who function as primary caregivers.	BabyWatch
Community, social and civic life	Socialising and volunteering includes: engaging in charitable organizations, service clubs or professional social organizations, informal and formal associations; etc.	Momento Events Tracker

The remaining SQS (N=22: 42Goals, AskMeEvery, ChartMySelf, DailyBurn, Daytum, Garmin MyConnect, Google Forms, KeepTrack, Lift, Reporter, and self-created tracker application, Dacadoo, Digifit, Fluxtream BodyTrack, Notch.Me, Open.Sen.Se, self-created application, Statwing, Tictrac, TRAQS.me, Zenobase, and Zoomdata) were found to capture a range of health aspects that belong to more than one health domain as denoted by the CDA-SQS; thus for the sake of clarity, they were listed in text but not presented in the above table.
